# Chronic Organic Magnesium Supplementation Enhances Tissue-Specific Bioavailability and Functional Capacity in Rats: A Focus on Brain, Muscle, and Vascular Health

**DOI:** 10.1007/s12011-025-04678-y

**Published:** 2025-06-04

**Authors:** Basar Koc, Ferda Hosgorler, Sevim Kandis, Burcu Acikgoz, Servet Kizildag, Ozge Guner, Nergiz Durmus, Mehmet Ates, Nazan Uysal

**Affiliations:** 1 Department of Physiology, School of Medicine, Dokuz Eylül University, Izmir, Türkiye; 2 Department of Pharmacology, Medical School, Dokuz Eylul University, Izmir, Türkiye; 3 College of Vocational School of Health Services, School of Medicine, Dokuz Eylül University, Izmir, Türkiye

**Keywords:** Magnesium supplementation, Magnesium bioavailability, Cognitive function, Neuromuscular performance, Learning and memory

## Abstract

Magnesium (Mg) is crucial in numerous physiological functions, including neuromuscular activity, energy metabolism, and cognitive processes. Despite its significance, the bioavailability and functional impact of different Mg formulations remain underexplored. This study investigates the long-term effects of chronic organic Mg supplementation (citrate, glycinate, malate) on tissue-specific Mg distribution and functional outcomes in rats. Thirty-eight adult Sprague Dawley rats were allocated into control and Mg-supplemented groups, receiving 35.4 mg/kg/day of elemental Mg for 8 weeks. Cognitive and behavioral assessments were conducted to evaluate learning, memory, and anxiety-like behavior, including the Morris water maze, open-field test, and elevated plus maze. Neuromuscular function was assessed via the grip strength and rotarod performance tests. Biochemical analyses of brain regions, skeletal muscle, and vascular tissue were performed to determine Mg levels, brain-derived neurotrophic factor (BDNF), and corticosterone concentrations. Results demonstrated that Mg-malate supplementation significantly increased Mg levels in skeletal muscle and whole-brain tissue, correlating with enhanced neuromuscular performance. Mg-citrate selectively elevated hippocampal BDNF levels, improving spatial learning and memory, while Mg-glycinate exhibited anxiolytic properties by reducing thigmotaxis behavior. Interestingly, despite increased aortic Mg levels, vascular relaxation responses were diminished in Mg-malate and Mg-citrate groups, suggesting a complex interplay between Mg accumulation and vascular reactivity. These findings highlight the formulation-dependent bioavailability and functional effects of Mg, emphasizing the necessity of targeted supplementation strategies for neurological, muscular, and cardiovascular health. Further clinical studies are warranted to validate these effects in human populations.

## Introduction

Magnesium (Mg) is our planet’s eighth most common element [1]. It is also the fourth most abundant element in vertebrates and the second most common element in the cell, following potassium in eukaryotic cells. The human body has an average of 0.4 g/kg (total of 24 g in a 70-kg adult) of Mg [2]. Mg is a vital cell component in many physiological functions [2–4].

ATP metabolism is essential for muscle contraction and relaxation, normal neurological function, and release of neurotransmitters. Mg affects muscle performance through its fundamental role in muscle contraction/relaxation and energy metabolism [4, 5].

The suppressing effect of Mg on sensory or motor function and the conduction in excitable cells is a particularly important feature utilized in many treatment areas. It is administered for general use, such as pain palliation, tocolysis, treatment of eclampsia and preeclampsia, cardiac arrhythmias, anxiolysis, and as an antidepressant [6].

Inadequate Mg intake is associated with various diseases and symptoms [6–8]. Neuronal Mg concentration is critical in regulating NMDA receptor activation, which is essential for synaptic transmission, learning, and memory processes [9]. Experimental animal studies have demonstrated that Mg deficiency correlates with anxiety- and depression-like behaviors; however, human studies investigating its therapeutic potential remain limited. Furthermore, the form of Mg significantly impacts its absorption: inorganic Mg salts (e.g., Mg-oxide), while containing high elemental Mg, exhibit lower bioavailability compared to organic salts (e.g., Mg-citrate) due to their poor solubility [10].

Current understanding of the bioavailability and efficacy of various Mg compounds remains limited. We postulated that organic Mg compounds (e.g., citrate, glycinate, malate) demonstrate tissue-specific bioavailability and differential functional impacts on the brain, muscle, and behavioral outcomes. This study investigates the tissue-specific accumulation and functional effects of chronic supplementation with organic Mg formulations. We hypothesized that these compounds differentially impact Mg levels in the brain, skeletal muscle, and vasculature, influencing behavioral, cognitive, and motor outcomes. To address this, we assessed:

1. Tissue-specific Mg accumulation in brain, muscle, and vascular tissues.
2. Cognitive function via learning and memory tests.
3. Behavioral phenotypes related to anxiety.
4. Neuromuscular performance parameters.

## Materials and Methods

Thirty-eight adult outbred Sprague Dawley rats (Dokuz Eylul University School of Medicine, Experimental Animal Laboratory, Izmir, Turkey) were used in this study. All rats were housed in individual cages with free access to water and standard laboratory chow (Optima rodent 20, Bolu, Turkey). They were kept in a 12-h light/12-h dark cycle at constant room temperature (22 ± 1 °C) and humidity (60%). Dokuz Eylul University School of Medicine, Animal Care Committee, with 36/2018 protocol number, approved all experimental procedures.

### Experimental Groups

Rats were randomly assigned to four groups:

- Control (*n* = 8): received tap water.
- Mg—citrate (*n* = 10): 35.4 mg/kg/day.
- Mg—glycinate (*n* = 10): 35.4 mg/kg/day.
- Mg—malate (*n* = 10): 35.4 mg/kg/day.

Mg formulations were dissolved in tap water and administered via oral gavage for eight weeks.

### Experimental Design

**Fig. 1:**
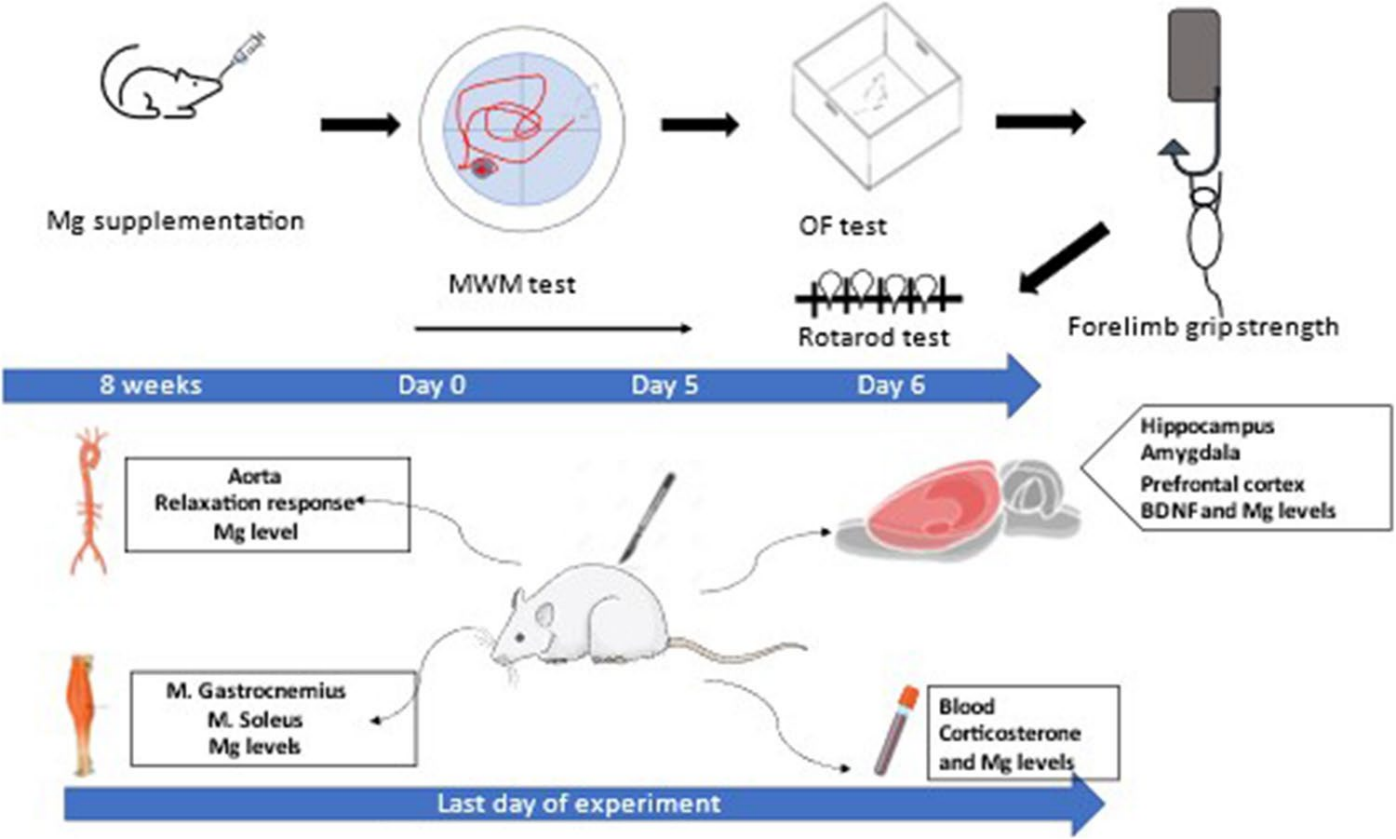
Experimental flow diagram

Mg-citrate, Mg-glycinate, and Mg-malate were dissolved in tap water at an elemental Mg dose of 35.4 mg/kg/day and administered orally through an oral gavage pipette as a single dose per day for 8 weeks (approximately 2.5 years in humans) [11–13]. The control group was given the same volume of tap water orally through an oral gavage pipette. All Mg compounds were presented in 1 ml volume/for each rat. We looked at different tissues to assess the bioavailability of Mg. We did some behavioral tests to measure the effects of Mg on the brain, the open-field test, and the elevated plus maze test to estimate parameters of emotional behavior. We used the Morris Swimming Test to evaluate learning and memory. The Noldus Ethovision XT video-tracking system completed the recording and analyses. To assess the effects of Mg on skeletal muscle tissue, we assessed locomotor activity, muscle strength levels, and motor coordination. An isolated tissue bath was used to evaluate the smooth muscle (Fig. 1).

### Assessment of Behavioral Experiments

#### Open-Field Test

The rodents’ locomotor activity, exploratory behavior, and emotional behavior were evaluated in the open-field test [14]. A black plexiglass box with a height of 50 cm and dimensions of 100 × 100 cm was used. The box floor was divided into equal squares (75% at the edges, 25% at the center) and illuminated with a light source (100 lx). The rats were placed in the center of the field, and the durations spent at the edges and in the center area of the field were recorded with the video tracking system for 300 s. This test recorded the total distance traveled, time spent in the center area, and margin areas. Increased amounts of time spent in the edges area or immobility were accepted as signs of anxiety. The Thigmotaxis zone (margin area) was defined as 5 cm from the arena walls [15].

#### Elevated Plus Maze Test

All animals performed the elevated plus maze test to assess their anxiety-like behavior. The height of the platform used for the elevated plus maze test was 50 cm. There is a square-shaped area of 10 × 10 cm in the center. It consists of two open arms (50 cm long and 10 cm wide, with a 1 cm high border) and two closed arms (50 cm long and 10 cm wide, with walls 50 cm high) extending from the edges of the center area. The subject was placed in the middle of the central platform, facing one of the open arms, and monitored with a camera for 300 s. The maze was cleaned with 70% ethanol between each trial. The total number of entries to arms and total time spent in arms were recorded via Noldus Ethovision XT software [16].

#### Morris Water Maze Test

Spatial learning and memory performance were tested in the Morris water maze. The Morris water tank is a black circular pool with a diameter of 140 cm and a depth of 75 cm, made of non-reflective material. The tank is filled with 22 °C water up to 50-cm level, and a cylindrical hidden platform with a diameter of 10 cm is placed hidden below the water level. Various colored and illuminated objects were placed around the environment where the pool was located to be used as clues. The Morris water tank was divided into four equal quadrants, southeast (SE), southwest (SW), northeast (NE), and northwest (NW), by two lines that intersect each other right in the middle of the tank, forming a cross shape. The points where the plus shape cut the tank’s walls were determined as north, south, east, and west. The platform was placed in the southwest quadrant of the tank, 1–2 cm below the water surface. Its position was not changed during the learning trials. The subjects were placed into the water from a different point each day, facing the tank’s walls. After placing the animal into the pool, the subject was expected to find the platform within 60 s. If the subject could not find the hidden platform within 60 s, it was placed on the hidden platform, and the animal then observed the environment for 30 s. Learning trials with this method continued for four days. On the fifth day, a probe trial was performed on each animal. In the probe trial, the platform was removed from the tank, allowing the subject to swim freely for 60 s. The time spent in the SW quadrant, where the platform is located in the previous days, was evaluated as spatial memory performance. The behavioral data of the subjects were assessed using the Noldus Ethovision video tracking system. The total swimming distance in each quadrant, time to reach the platform, and time spent in each quadrant were recorded [17, 18].

#### Forelimb Grip Strength Test

The subjects’ muscle strength was measured using a muscle strength meter, Sundoo SH-50 (Wenzhou Sundoo Instruments Co., Wenzhou, China). The rats were lifted from their tails to grip the device bar with their forearms and gently pulled after grasping the bar. The maximum value the digital force indicator provided before releasing the bar was defined as the grip force. The test was repeated thrice in a row, and the mean value of the holding force was considered. [19].

#### Rotarod Performance Test

The rotarod test device comprises a 6-cm diameter rotating rod, fall sensors, latency-to-fall timers, and separator panels. The speed of the rod was linearly increased from 4 to 40 rpm for 180 s. The animals were trained to walk on the accelerating rotarod on the first day. Motor performance measurements were made just once after 8 weeks of Mg supplementation. The counter timed each walking performance on the rotarod until the animal lost balance and fell down. The last rpm at which the animals could walk was evaluated as the motor performance of the animals [19].

### Isolated Tissue Bath

#### Preparation of Tissues

Aortic rings 2–3 mm in length were prepared and mounted in 20-ml organ baths containing the Krebs solution of the following composition in (mM); Krebs (NaCl 133, KCl 5, CaCl_2_ 2.5, MgSO_4_ 1.2, NaHCO_3_ 12, NaH_2_ PO_4_ 1, glucose 11). The bath solution was maintained at 37 °C and constantly oxygenated with 95% O_2_ and 5% CO_2_. The preparations were allowed to equilibrate for at least 1 h under 1-g resting tension. During the 60-min rest period, tissues are exposed to Krebs solution and then washed with 60 ml of the solution at the end of 60 min to assess the viability of the tissues at the end of 60 min. Tissues were contracted with mM KCL. To evaluate smooth muscle contraction responses, tissues cumulatively with phenylephrine (10–9 between concentrations of −10–4 M) contractions were provided by trying. Thirty-minute rest period followed by smooth muscle to evaluate the relaxation responses to the EC80 dose of phenylephrine. Sodium nitroprusside (10–8 −10–5 M) (SNP) was applied. Data were transferred to the biopac mp system via the MAY transducer—the results of the contraction response of the tissues after phenylephrine was accepted as 100%. Relaxation responses were calculated separately for each dose of SNP, and the contraction responses to phenylephrine were expressed as %[20, 21].

#### Tissue Extraction and Homogenization of Tissues

Rats were gone under CO_2_ via cardiac puncture to achieve exsanguination before tissue harvesting. After exsanguination, all tissues (brain and regions such as hippocampus, prefrontal cortex, amygdala, uterus, aort, gastrocnemius muscle, and soleus muscle) were dissected. After the animals were euthanized via CO₂ inhalation followed by cardiac puncture to ensure exsanguination, dissection was initiated immediately to prevent post-mortem degradation. All procedures were performed under sterile conditions using autoclaved surgical instruments. First, the thoracic cavity was opened to extract the heart and aorta, followed by carefully separating the descending thoracic aorta using micro-scissors and forceps. Subsequently, the abdominal cavity was exposed to isolate skeletal muscles: gastrocnemius and soleus muscles were bilaterally excised. For brain extraction, a midline scalp incision was made, and the skull was opened using rongeurs. The brain was gently removed en bloc and placed on a chilled dissection platform. The whole brain was sectioned coronally to isolate the hippocampus, prefrontal cortex, and amygdala using fine micro-dissecting tools, guided by a rat brain atlas (Paxinos & Watson). All tissues were immediately snap-frozen in liquid nitrogen and stored at − 85 °C until biochemical analyses. Then, the tissue samples stored at − 85 °C were homogenized in iced cold PBS (HyClone Phosphate Buffered Saline solution, cat no: SH30256.01) with BioSpec mini beadbeater-16, 60 Hz, and 3450 oscillation/min. After homogenization, the homogenates were centrifuged for 15 min at 10,000* g* at 4 °C. The supernatants of all brain tissues were used to measure BDNF (Catalog Number EK0308, BosterBio, Wuhan, China) via the ELISA method. Blood serum was used to analyze corticosterone levels (Elabscience Rat Corticosterone, Wuhan, China) via the ELISA method. Protein analyses of tissue homogenates were performed according to the manufacturer’s description of the Thermo Scientific™ Pierce™ BCA Protein Assay kit (Cat No 23227, Thermo Fisher Scientific, USA). The results were expressed as BDNF per mg tissue protein. The absorbency changes were measured with a microplate reader (ELx800, BioTek Instruments, Inc., Winooski, VT, USA) at 450 nm for the ELISA kit and 560 nm for the protein assay kit. Serum Mg levels were quantified using a Beckman Coulter AU5800 biochemistry analyzer via colorimetric assay with xylidyl blue. Results were expressed as mmol per 10 g tissue protein.

## Results

### Emotional Behavioral Parameters

In the elevated plus maze test performed for anxiety, there was no significant difference between the control and Mg groups.

**Fig. 2:**
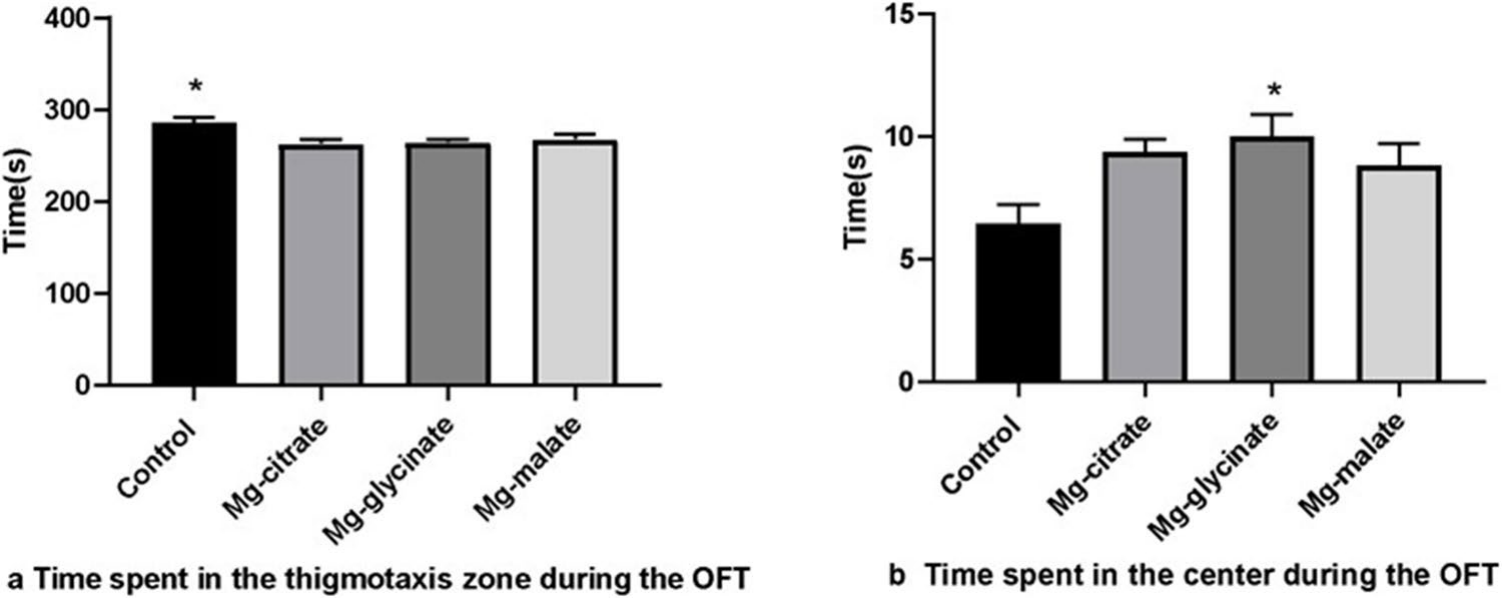
**a** Time spent in the thigmotaxis zone during OFT. **b** Time spent in the center during the OFT

In the open-field test, the control group’s thigmotaxis time was longer than all Mg groups (*p* < 0.05) (Fig. 2a). Also, the Mg-glycinate group demonstrated more activity in the center of the open-field arena than the control group (*p* < 0.05) (Fig. 2b). No significant difference was found among the Mg-supplemented groups in center time or thigmotaxis behavior.

No significant difference was observed between the magnesium-treated groups in terms of center exploration or thigmotaxis, indicating comparable anxiolytic potential across formulations.

### Learning and Memory

**Fig. 3:**
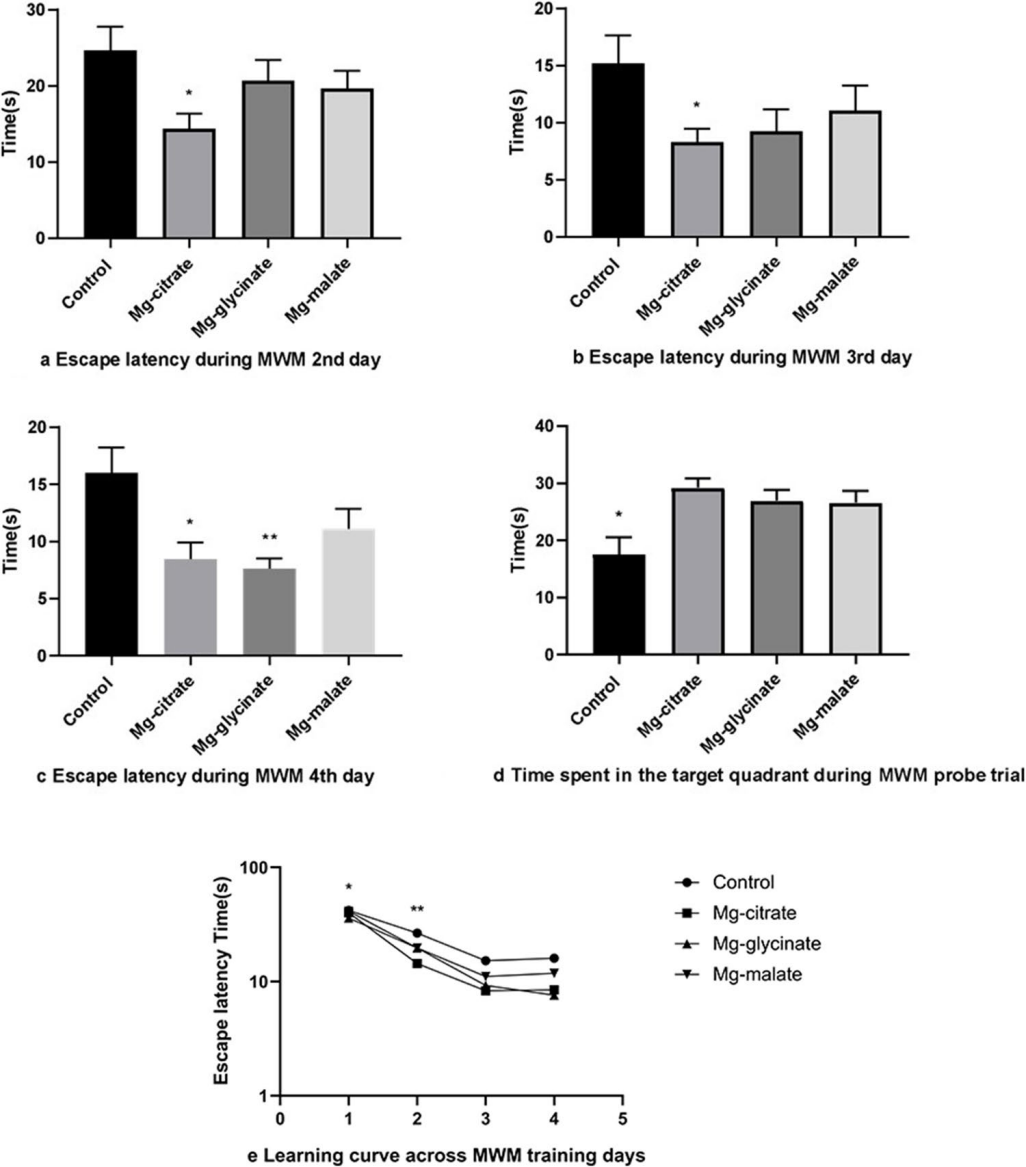
**a** Escape latency during MWM 2nd day. **b** Escape latency during MWM 3rd day. **c** Escape latency during MWM 4 th day. **d** Time spent in the target quadrant during MWM probe trial. **e** Learning curve across MWM training days

The Mg-citrate group showed shorter escape latency than the control group on the second and third days of learning trials (*p* < 0.05) (Fig. 3a, b). On the fourth day of the learning trial, escape latencies were lower in the Mg-citrate and Mg-glycinate groups (*p* < 0.05) (Fig. 3c).

In probe trials, time spent in the target quadrant was used to evaluate long-term memory. Mg-citrate, Mg-glycinate, and Mg-malate spent more time in the target quadrant than control group animals (*p* < 0.05) (Fig. 3d).

Intragroup comparisons were performed across days using repeated-measures ANOVA, and the results are summarized in Fig. 3e. A significant reduction in escape latency was observed over days within each group. Specifically, the control group’s first-day escape latencies were significantly higher than those of the third (*p* = 0.005) and fourth (*p* = 0.006) days.

The Mg-citrate group showed a consistent improvement, with the first day significantly different from the second (*p* = 0.001), third (*p* < 0.001), and fourth (*p* < 0.001) days.

Similarly, the Mg-glycinate group demonstrated significant improvements, with first day higher than the second (*p* < 0.05), third (*p* < 0.001), and fourth (*p* = 0.001) days. Additionally, second-day latency was higher than the third (*p* = 0.007) and fourth (*p* = 0.004) days.

In the Mg-malate group, first-day latencies were significantly higher than second (*p* < 0.05), third (*p* = 0.003), and fourth (*p* = 0.004) days. These findings indicate successful spatial learning acquisition within each group across the trial days.

### Motor Performance Parameters

In the forelimb grip strength test for muscle strength comparison,

* Mg-malate group’s (12.08 ± 0.3395) muscle strength was more than other groups *p* < 0.001;

** Mg-citrate group’s (3.661 ± 0.1922) muscle strength was higher than control group’s (9.200 ± 0.4884) muscle strength *p* < 0.0001. Mg-citrate group’s (3.661 ± 0.1922) muscle strength was higher than Mg-glycinate group’s (2.221 ± 0.07467) muscle strength *p* < 0.05.

**Fig. 4:**
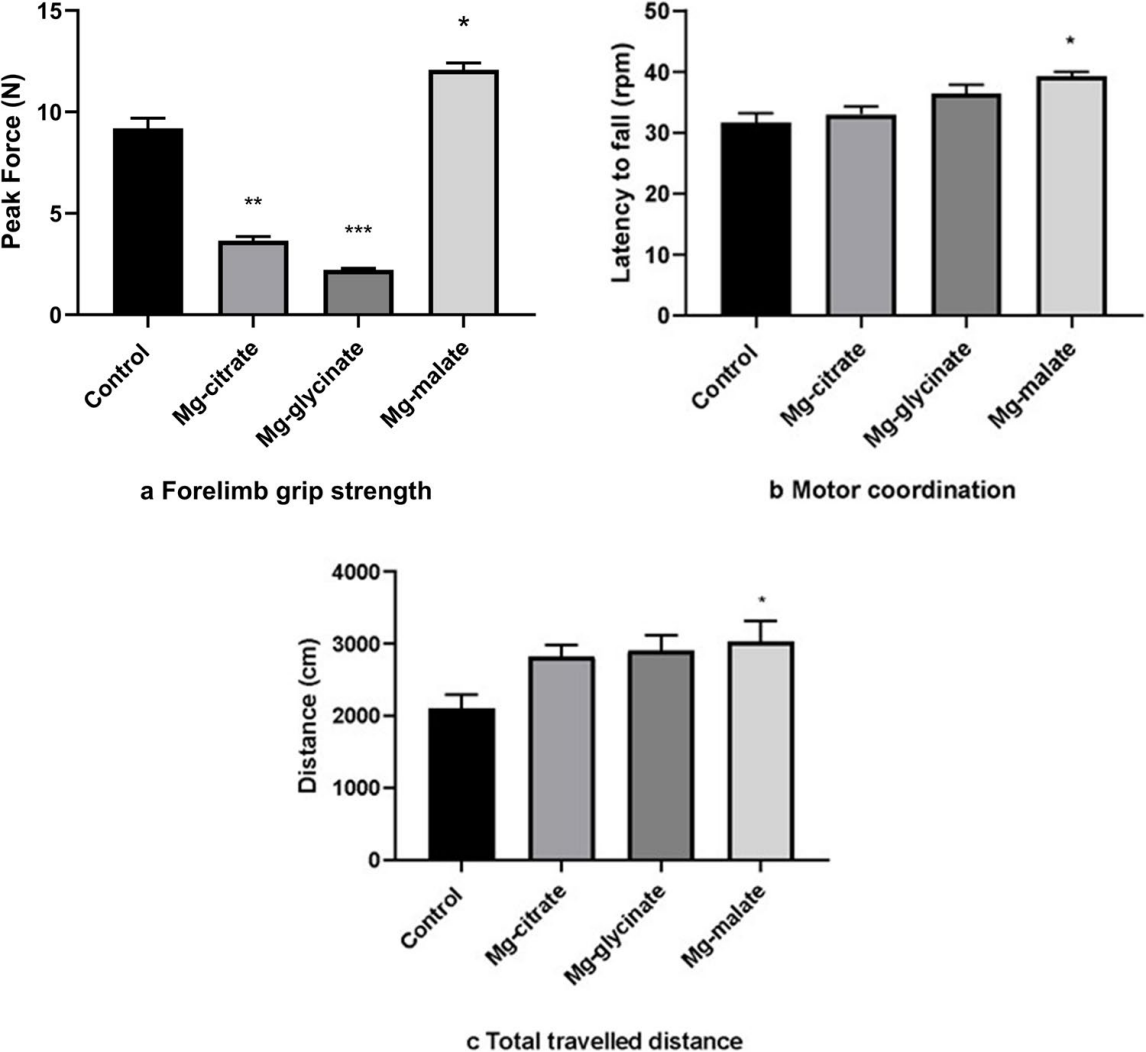
**a** Forelimb grip strength. **b** Moto coordination. **c** Total travelled distance

*** Mg-glycinate group’s (2.221 ± 0.07467) was higher than control group’s (9.200 ± 0.4884) muscle strength *p* < 0.0001 (Fig. 4a).

In the rotarod test, the Mg-malate group’s rotarod performance was higher than the control group’s (*p* < 0.05) (Fig. 4b). In the open-field test, Mg-malate group’s total distance traveled was more than the control group’s total distance (*p* < 0.05) (Fig. 4c).

However, there were no statistically significant differences between the Mg-malate group and the other magnesium-treated groups (Mg-citrate and Mg-glycinate). This suggests that while Mg-malate increases locomotor activity compared to baseline (control), its superiority over other magnesium compounds in this regard was not statistically confirmed.

**Fig. 5:**
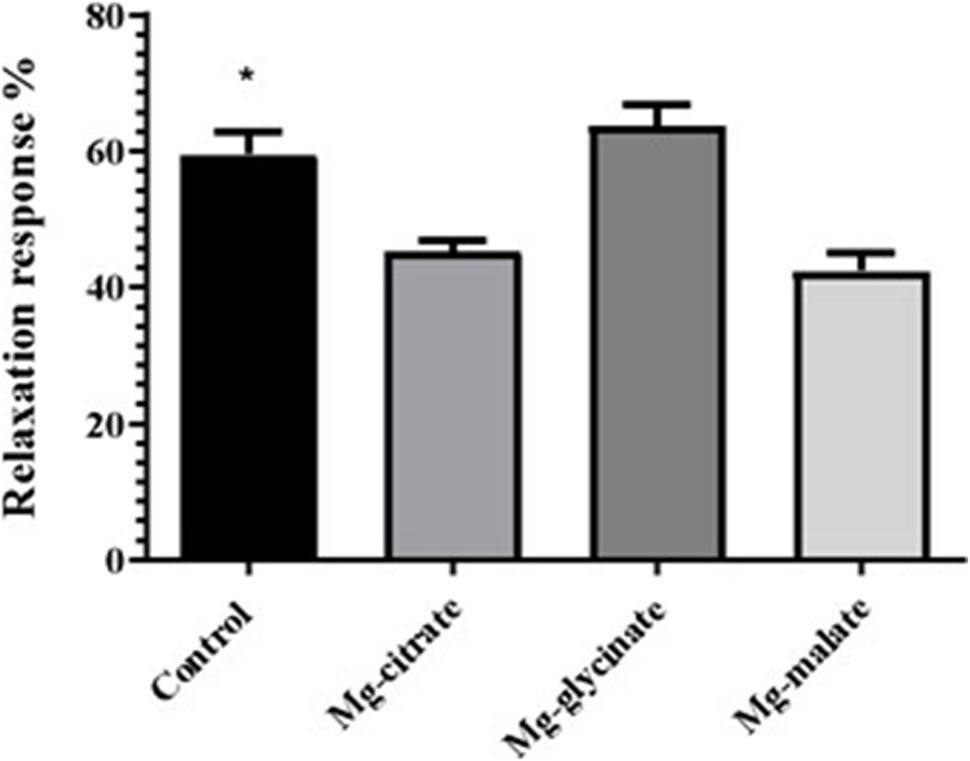
Endothelium-independent relaxation response at 10^−8^ m

Endothelium-independent relaxation responses at 10^−8^ M, Mg-malate, and Mg-citrate groups were significantly lower than the control group (*p* < 0.05) (Fig. 5).

### Biochemical Parameters

#### Tissue-Specific Mg Accumulation

**Fig. 6:**
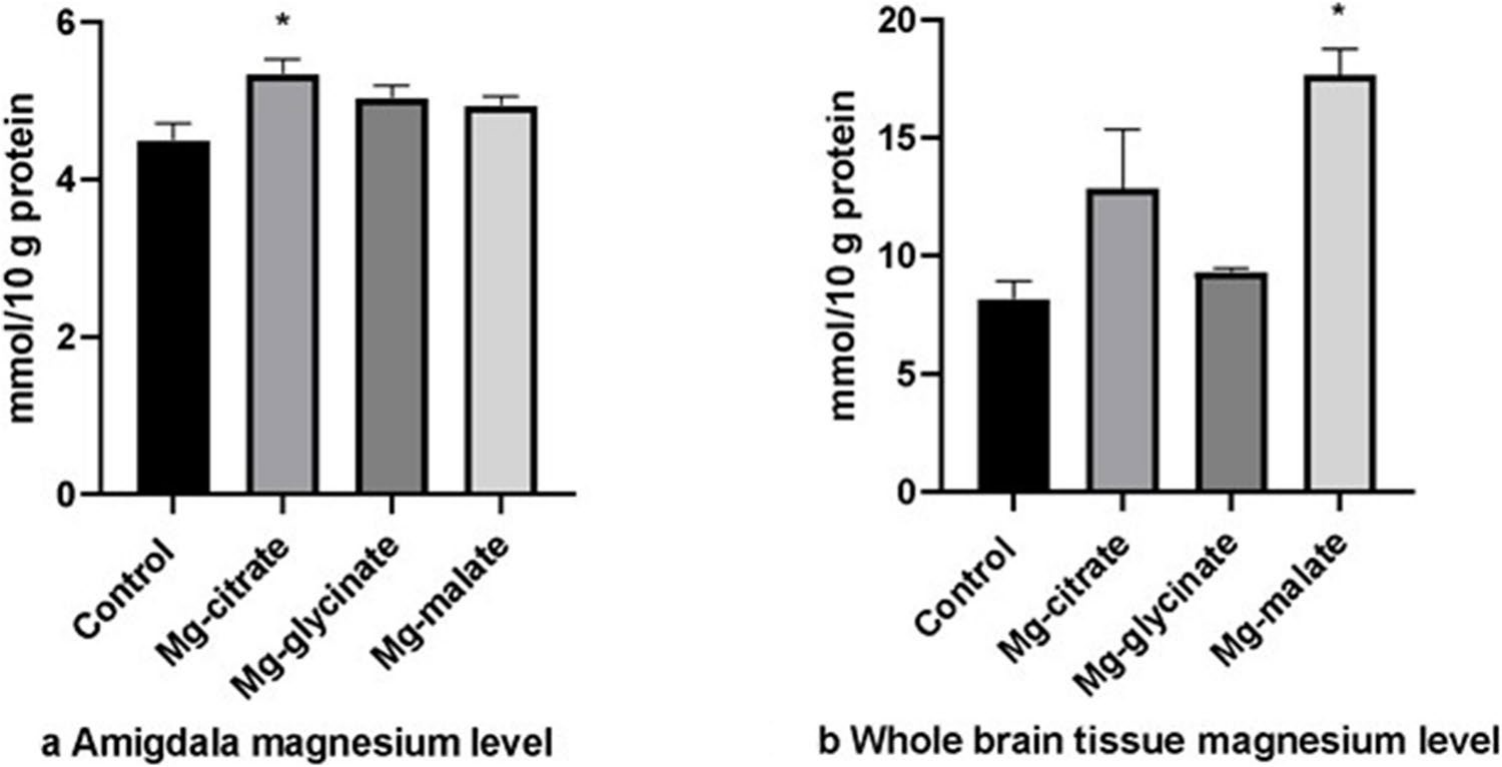
**a** Amigdala magnesium level. **b** Whole brain tissue magnesium level

The Mg citrate group’s amygdala Mg level is higher than the control group’s (*p* < 0.05) (Fig. 6a). While Mg-citrate significantly increased amygdala Mg vs control, no differences were observed among the three Mg-treated groups. The Mg-citrate group had an amygdala Mg concentration of 5.343 ± 0.1877 mmol/10 g protein, which was significantly higher than the control group’s value of 4.495 ± 0.2278 mmol/10 g protein (*p* < 0.05). However, there were no statistically significant differences among the magnesium-treated groups in amygdala Mg levels, suggesting similar regional accumulation profiles.

The Mg-malate group’s whole-brain Mg level is higher than the control group (*p* < 0.05) (Fig. 6b). There were no significant differences in the Mg level of other brain tissues.

**Fig. 7:**
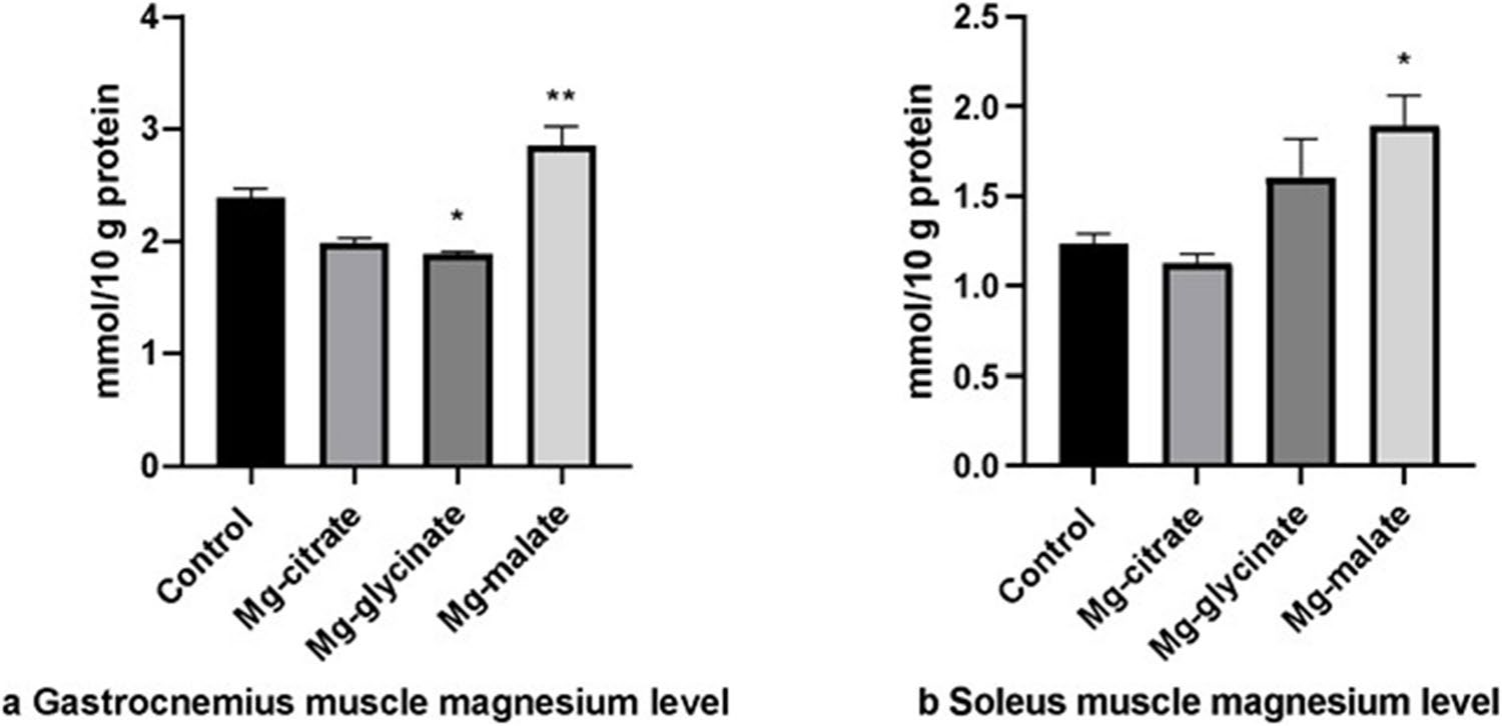
**a** Gastrocnemius muscle magnesium level. **b** Soleus muscle magnesium level

The Mg levels in the gastrocnemius muscle were significantly higher in the Mg-malate group than in the other groups (*p* < 0.05) (Fig. 7a). Conversely, the Mg level in the Mg-glycinate group was lower than in the control and Mg-malate groups (*p* < 0.05) (Fig. 7a). Additionally, Mg levels in the soleus muscle were higher in the Mg-malate group than in the control group (*p* < 0.05) (Fig. 7b).

**Fig. 8:**
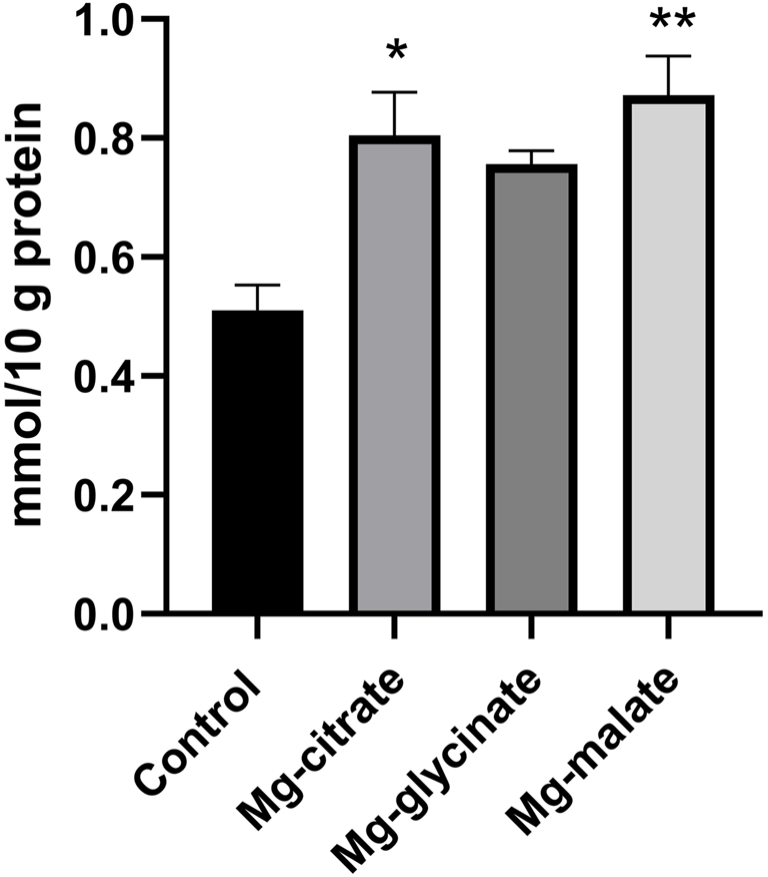
Arcus aorta magnesium level

Magnesium level of aorta; * Mg-citrate group Mg levels (0.8046 ± 0.07255) was higher than control group (0.5103 ± 0.04268) *p* = 0.0039 ** Mg-malate group’s Mg levels (0.8724 ± 0.06564) was higher than control group (0.5103 ± 0.04268) *p* = 0.0039 (Fig. 8). There were no statistically significant differences between magnesium groups.

#### BDNF and Corticosterone Levels

**Fig. 9:**
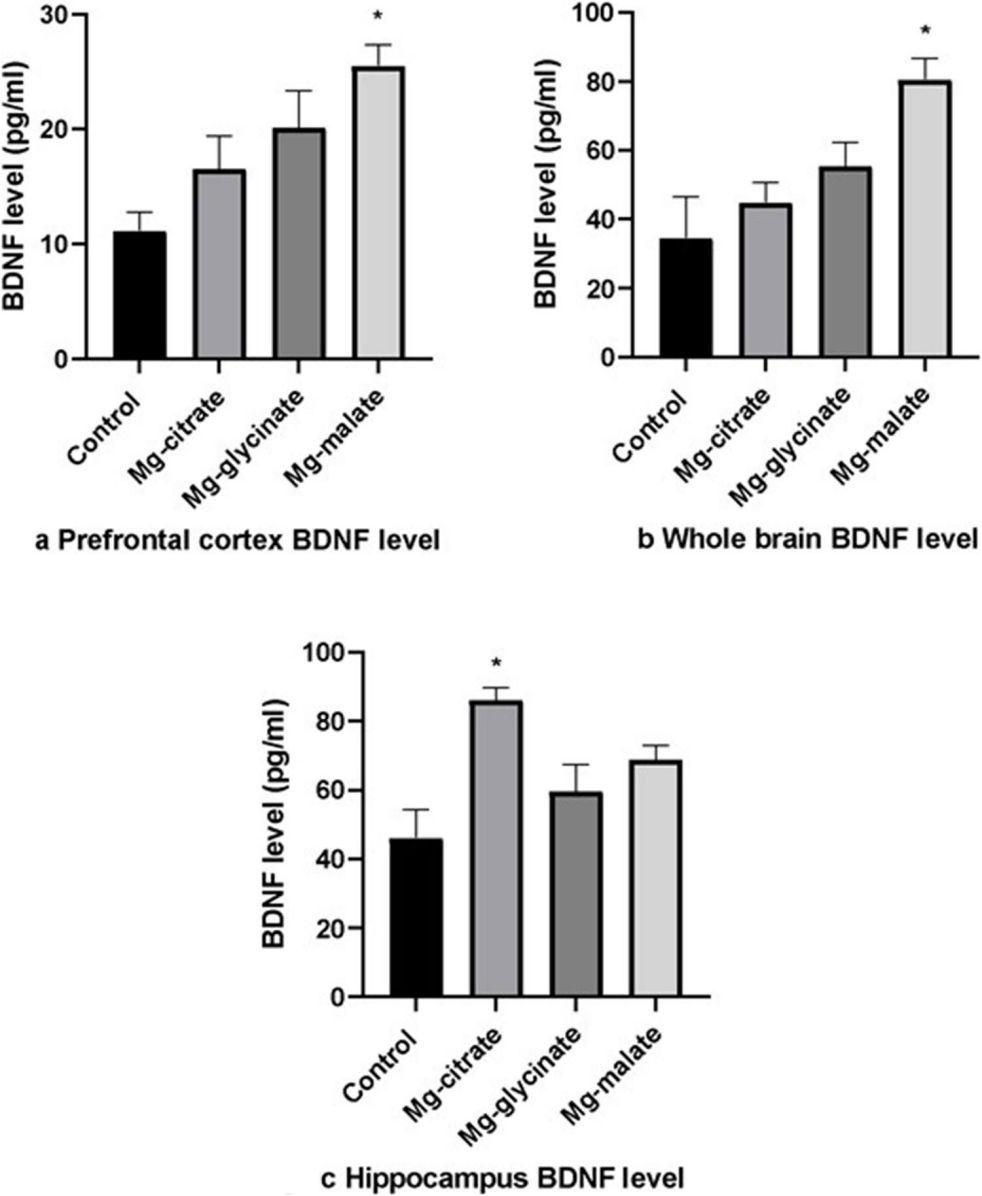
**a** Prefrontal cortex BDNF level. **b** Whole brain BDNF level. **c** Hippocampus BDNF level

Prefrontal cortex and whole-brain BDNF levels were higher in the Mg-malate group than in the control group (*p* < 0.05) (Fig. 9a, b). Whole-brain BDNF levels were 80.59 ± 6.18 pg/mg in the Mg-malate group, significantly higher than the control group’s 34.62 ± 11.92 pg/mg (*p* < 0.05). While Mg-malate showed the most robust increase, no statistically significant differences in BDNF levels were observed between the Mg-citrate and Mg-glycinate groups, indicating that all formulations had some capacity to enhance neurotrophic signaling. Hippocampus BDNF levels were higher in the Mg citrate group than in the control group (*p* < 0.05) (Fig. 9c). The Mg-citrate group showed a hippocampal BDNF concentration of 86.20 ± 3.62 pg/mg protein, compared to 46.21 ± 8.29 pg/mg in the control group (*p* < 0.05).

While each Mg group showed benefits versus control in different brain regions, there were no statistically significant differences among the Mg-treated groups regarding BDNF levels.

**Fig. 10:**
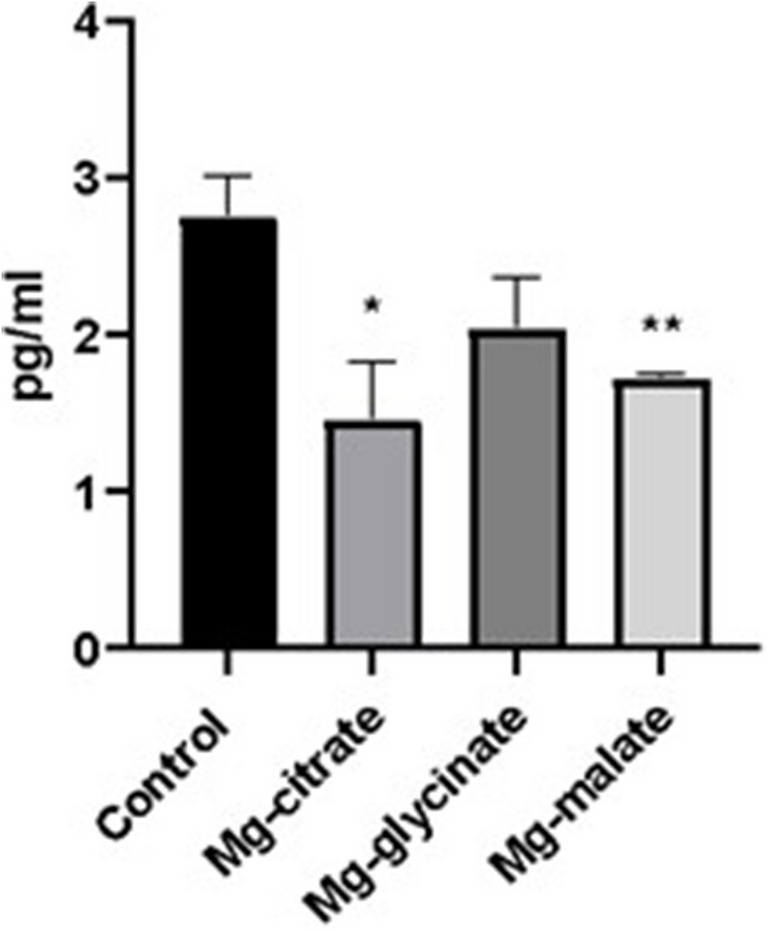
Blood corticosterone level

Blood corticosterone levels of Mg-malate and Mg-citrate were lower than the control group (*p* < 0.05) (Fig. 10).

### Statistical Analysis

Statistical analyses were conducted as follows: learning experiments were evaluated using two-way repeated-measures ANOVA, while other experiments were analyzed with one-way ANOVA. Post hoc pairwise comparisons were performed with Tukey’s test, and all analyses were carried out in GraphPad Prism 8.0. All data are presented as mean ± SEM. A significance threshold of *p* < 0.05 was applied throughout the study. Inter-group comparisons among the three magnesium-treated groups were additionally performed for behavioral, biochemical, and Mg distribution outcomes.

## Discussion

This study demonstrates formulation-specific effects of 8-week oral administration of organic Mg compounds (citrate, glycinate, malate) on Mg levels in the brain, skeletal muscle, and smooth muscle and behavioral outcomes in rats. The findings indicate that Mg’s bioavailability and functional efficacy are influenced by its chemical form and tissue-specific accumulation patterns. These results align with existing evidence highlighting Mg’s role in neurological, muscular, and vascular health while providing novel insights into the long-term benefits of organic Mg formulations. Notably, the observed differences in tissue accumulation and behavioral outcomes (e.g., anxiety, grip force, learning, and memory) suggest that Mg’s mechanisms of action are intrinsically linked to its chemical structure and tissue-targeted distribution.

### Biochemical Findings: Tissue-Specific Mg Accumulation and Stress Modulation

Mg Distribution: The differential tissue accumulation of Mg-citrate (amygdala) and Mg-malate (brain and skeletal muscle) likely reflects their divergent pharmacokinetic properties, including absorption kinetics and tissue-specific affinity. The lipophilic nature of Mg-malate may enhance passive diffusion across intestinal membranes, whereas the high water solubility of Mg-citrate could promote paracellular transport via tight junctions [22, 23]. Notably, Mg-malate induced BDNF elevation in the prefrontal cortex aligns with its putative role in enhancing executive function and stress resilience, while Mg-citrate’s selective upregulation of hippocampal BDNF mirrors its efficacy in mitigating age-related cognitive decline, as demonstrated in preclinical aging models [24–26].

### Behavioral and Emotional Consequences: Anxiolytic Potential of Mg

The open-field test was utilised as a primary research tool, with the results indicating a significant reduction in anxiety-like behavior, as indicated by reduced thigmotaxis and increased center activity, particularly in the Mg-glycinate group. This finding is consistent with the findings of numerous studies that have reported anxiolytic effects of Mg. For instance, rats given high Mg in drinking water exhibited significantly reduced anxiety (as measured by elevated plus maze and open-field tests) without any motor impairment. From a mechanistic perspective, Mg’s NMDA receptor blockade and GABAergic modulation have the capacity to attenuate excitatory stress circuits. It is noteworthy that Mg-citrate increased amygdala Mg levels (Fig. 6a). The amygdala has been identified as a central region in the processing of fear and anxiety. Therefore, elevated Mg levels in this region could be a contributing factor to reduced anxiety, although this hypothesis requires further investigation. In a similar manner, the increase in brain magnesium (whole brain and prefrontal cortex) resulting from magnesium malate administration may engender global calming effects. The present findings suggest that different Mg salts may preferentially target anxiety circuits: The marked effect of Mg-glycinate on central activity may be partly due to glycine itself (as a neurotransmitter) or efficient brain uptake via amino-acid transporters. In summary, the anxiolytic behavioral outcomes reported here are consistent with those documented in previous literature, thereby supporting Mg’s ability to blunt stress responses. Future studies could directly assess amygdala and hippocampal activity, or utilise stress-induced models to dissect these form-specific effects [27–30].

The reduced thigmotaxis duration in Mg-treated groups during the open-field test, alongside increased central area activity in the Mg-glycinate group, demonstrates that chronic Mg supplementation modulates anxiety-related behavioral responses. These findings align with prior evidence indicating Mg’s ability to attenuate stress responses via suppression of the hypothalamic–pituitary–adrenal (HPA) axis [27, 29, 31, 32]. However, the absence of significant differences in the elevated plus maze test implies that Mg’s anxiolytic effects may be context- or stressor-dependent, highlighting the importance of methodological variables in interpreting anxiety-related outcomes.

The administration of Mg-malate and Mg-citrate resulted in a significant reduction in plasma corticosterone levels, suggesting a dampened activity of the HPA axis. This finding extends the evidence that adequate Mg status has a role in the regulation of stress hormones. The administration of low-magnesium (Mg) diets to rodents has been demonstrated to induce hyperactivity of the hypothalamic–pituitary–testicular axis (HPA) and elevate corticosterone levels. However, the replenishment of Mg has been shown to restore HPA axis function to a normal state. The reduction in corticosterone is hypothesised to underlie the improved behavior and increased BDNF. Glucocorticoids have been demonstrated to suppress BDNF expression, and thus a reduction in corticosterone would be expected to free neurotrophic signalling. In line with this, other studies have reported that Mg deficiency elevates cortisol/corticosterone and anxiety, and that Mg supplementation reverses these effects. The present data thus suggest that Mg acts indirectly via stress-hormone pathways: chronic Mg may inhibit adrenal release of glucocorticoids or feedback more efficiently through the hippocampus. The endocrine implications of this phenomenon, however, were not sufficiently emphasized in the discussion. Nonetheless, they align with the findings of human research that demonstrates that restoring magnesium (Mg) levels can reduce circulating cortisol and enhance stress resilience. Further research is required to measure ACTH or CRH levels in order to ascertain the precise location of Mg’s action within the HPA axis [27, 29, 33–35].

### Motor Performance: Superiority of Mg-Malate on Muscle Bioavailability and Energy Metabolism

The administration of Mg-malate resulted in a significant increase in muscle Mg levels, concurrently engendering optimal levels of grip strength and rotarod performance. This provides evidence that supports Mg’s essential role in muscle bioenergetics. It is hypothesized that malate (an intermediate in the Krebs cycle) facilitated ATP production, thereby improving muscle function. Indeed, prior research demonstrates that Mg supplementation enhances muscle regeneration and strength. For instance, mice administered MgSO₄ (50 mg/kg) exhibited augmented myofiber dimensions and enhanced grip strength. Conversely, Mg-glycinate resulted in a surprising decrease in muscle Mg, even falling below the control group, despite the observation of modest performance gains. This phenomenon may be indicative of disparate absorption or metabolism of the glycine complex. Amino-acid-bound Mg can be transported via peptide carriers and may distribute differently (even being used for glycine supply). Further research is required to ascertain the exact nature of this distribution. Conversely, the lipophilicity of Mg-malate is hypothesised to have enhanced muscle uptake, thereby corroborating the established correlation between elevated Mg levels and augmented muscle strength, as reported in the study. Mg-malate and Mg-glycinate have been shown to enhance grip strength compared to the control group. This finding is consistent with the results of human and animal studies that have indicated that Mg repletion is associated with reduced muscle weakness. Together, these data reinforce the conclusion that organic Mg forms can bolster muscular performance, but muscle Mg accumulation is form-dependent. Future research could involve the measurement of muscle mitochondrial function and oxidative stress markers, to elucidate the manner in which each form contributes to muscle energetics [36–44].

### Cognitive Enhancement: Hippocampal Plasticity and BDNF Dynamics

Mg-citrate and Mg-glycinate improved spatial learning and long-term memory, correlating with elevated hippocampal BDNF and reduced corticosterone. The shorter escape latency in Mg-citrate groups during early learning trials (days 2–3 of MWM) suggests rapid synaptic remodeling [6, 24, 45, 46].

The observed increase in hippocampal BDNF levels in Mg-citrate-treated groups, despite unchanged Mg concentrations in the hippocampus, may arise from systemic or indirect neural mechanisms rather than direct local Mg accumulation. First, Mg-citrate reduced systemic stress and inflammation by modulating the hypothalamic–pituitary–adrenal (HPA) axis and suppressing pro-inflammatory cytokines (e.g., IL-6, TNF-α), both of which are known to inhibit BDNF expression [47–50]. Alleviating neuroinflammatory cascades and glutamate-induced excitotoxicity via NMDA receptor blockade [51] Mg may create a microenvironment conducive to BDNF synthesis without requiring elevated hippocampal Mg. Second, prefrontal-hippocampal circuit interactions could mediate this effect: higher Mg bioavailability in the prefrontal cortex (PFC) may enhance PFC-derived BDNF, which projects to and modulates hippocampal plasticity[24, 50, 52]. Third, gut-brain axis signaling might contribute, as Mg-citrate’s potential to improve gut microbiota composition could increase circulating short-chain fatty acids (e.g., butyrate), which cross the blood–brain barrier to stimulate BDNF production. These mechanisms collectively suggest that Mg-citrate enhances BDNF through indirect, multi-system pathways, with regional variability potentially reflecting differences in receptor density, Mg retention capacity, or network connectivity rather than localized Mg accumulation. Although hippocampal Mg levels showed a numerical increase in the Mg-citrate group compared to controls, the difference did not reach statistical significance. This discrepancy could stem from methodological limitations in detecting subtle, region-specific Mg changes. Interestingly, the higher levels of BDNF in the prefrontal cortex in the Mg-citrate group emphasize the differential bioavailability of Mg across brain regions [11, 15, 53–59].

Specifically, Slutsky et al. [24] demonstrated that increasing brain magnesium content via supplementation improved synaptic plasticity and spatial learning in rodents, supporting our findings of enhanced memory performance and increased hippocampal BDNF in the Mg-citrate group. Similarly, Abumaria et al. [25] showed that elevated brain magnesium facilitated fear extinction and modulated synaptic function in the prefrontal cortex and amygdala. This parallels the anxiolytic-like behavior and improved motor function observed in our Mg-malate group. These studies collectively support the notion that different magnesium formulations exert region-specific effects on brain function, consistent with the tissue-specific BDNF and Mg accumulation patterns we observed. Furthermore, our work builds on this literature by extending findings to skeletal muscle and vascular tissue, domains less often studied in magnesium supplementation research [24, 25].

In addition to the observed improvements in behavioral, neuromuscular, and cognitive parameters, it is essential to consider the role of oxidative stress as a potential mediator of magnesium’s effects. Magnesium has been shown to enhance antioxidant defense by stabilizing enzymatic systems such as superoxide dismutase (SOD), glutathione peroxidase, and catalase. Although oxidative stress markers were not assessed in the present study, it is plausible that the behavioral and biochemical benefits observed—including reduced corticosterone levels and elevated BDNF expression—may partly result from redox homeostasis improvements. Future studies incorporating oxidative stress markers could help clarify whether magnesium formulations’ neuroprotective and anxiolytic effects are mediated through antioxidant pathways, particularly in brain regions susceptible to oxidative damage, such as the hippocampus and prefrontal cortex [60, 61].

### Vascular Effects: The Paradox Between Mg Accumulation and Relaxation Responses

The unexpected finding that chronic Mg-malate and Mg-citrate increased aortic Mg content while reducing SNP-induced relaxation suggests adaptive vascular changes. Acutely, Mg is a well-known vasodilator (via Ca^2+^ antagonism and enhanced NO signaling). Indeed, many studies report that Mg supplementation improves endothelial function—for example, oral Mg raised flow-mediated dilation in humans. The present paradox may reflect chronic Mg-induced downregulation of relaxation pathways. Long-term Mg loading might reduce smooth muscle cell sensitivity to NO or alter Ca^2+^ channel expression, blunting relaxation. Similar paradoxical effects have been noted: chronic Mg can promote vascular remodeling and calcium handling adjustments in hypertension models. Our results thus align with a dual role for Mg in vasculature—acute Mg causes relaxation, but chronic Mg accumulation may lead to compensatory changes that diminish vasodilatory responses. Further studies should compare acute vs chronic Mg effects on vascular tone and examine the expression of endothelial NO synthase and ion channels for a mechanistic understanding [62–77].

### Limitations and Future Studies

Oral gavage stress: Although repeated gavage administration has minimal effect on stress biomarkers, it may be a potential confounding factor in the impact of Mg on behavioral outcomes. Mechanistic deficiencies: Tissue expression of Mg transporters such as TRPM6/7 and anion-Mg complex stability were not analyzed. Although atomic absorption spectroscopy would have provided more accurate intracellular magnesium measurements, resource limitations necessitated routine biochemical laboratory tests at the university hospital’s central laboratory. Only endpoint serum values were analyzed. Importantly, we considered the control group’s serum magnesium level the baseline reference, reflecting the physiological norm in untreated animals. Duration and human Validity: 8 weeks of administration equals ~ 2.5 years (lifespan scaling 1:12) relative to human lifespan in rats. Clinical studies are required to confirm formulation-dependent effects in humans. Comparative shortcomings: There is no direct comparison with Mg-L-threonate, whose cognitive effects are more popular. Also, future studies should integrate oxidative stress markers, such as SOD, MDA, and glutathione ratios, to further elucidate the antioxidant capacity of magnesium formulations and their potential role in mitigating cognitive and emotional impairments via redox modulation.

## Conclusion

This study provides critical information for clinical applications by revealing the tissue-specific bioavailability and functional effects of organic Mg formulations: Mg-malate may be favored in metabolic and neuromuscular disorders with muscle performance and whole-brain Mg/BDNF enhancement. Mg-citrate is advantageous in cognitive decline or anxiety states with hippocampal plasticity and stress resistance. Mg-glycinate has the potential for use in stress-related disorders by reducing anxiety-like behaviors.

These findings underscore the critical role of anionic composition in Mg supplementation for optimizing region-specific therapeutic outcomes. While preclinical models demonstrate formulation-dependent effects on brain Mg distribution and neuroplasticity, translating these results into clinical nutrition and therapeutic practice requires robust validation in human trials. Confirming these anion-specific mechanisms in humans will advance personalized supplementation strategies, bridging gaps between mechanistic research and interventions tailored to neurological or metabolic disorders.

## Data Availability

No datasets were generated or analysed during the current study.
